# Na^+^/K^+^-ATPase-dependent autophagy protects brain against ischemic injury

**DOI:** 10.1038/s41392-020-0153-7

**Published:** 2020-05-20

**Authors:** Mengyuan Zhu, Lei Cao, Siping Xiong, Haijian Sun, Zhiyuan Wu, Jin-Song Bian

**Affiliations:** 1 Department of Pharmacology, Yong Loo Lin School of Medicine, National University of Singapore, Singapore, 117600 Singapore; 2 National University of Singapore (Suzhou) Research Institute, Suzhou, China

**Keywords:** Cell biology, Diseases of the nervous system

**Dear Editor,**

Cerebral ischemic stroke is one of the leading causes of death worldwide with no effective treatment methods. Therefore, the investigation of potential intervention targets is urgently needed. Na^+^/K^+^-ATPase (NKA), a well-studied transmembrane protein pump expressed in all cells, is essential for the maintenance of cell membrane potential by exchanging three sodium ions out with two potassium ions into the cell to strictly regulate the electrochemical gradient and hence neuronal excitability. The energy demand of NKA-mediated maintenance of the membrane potential is ~40% of the energy produced by respiration in the brain.^1^ NKAα1 is ubiquitously expressed and important for ion gradient maintenance. Preservation of the function of NKAα1 was recently reported to relieve ischemic damage.^2^ A new role of NKA as signal transducer involving ligand–receptor interaction and activation of the non-receptor tyrosine kinase Src has been reported over the last 20 years.^3^ The autophagy pathway, a highly conserved self-eating catabolic pathway for the degradation of misfolded proteins or damaged organelles, is widely accepted to reduce neuronal injury when moderately activated during ischemia. The potential link between NKA and autophagy was defined and shown to require NKAα1 exclusively,^4^ but the specific mechanisms involved have not yet been clarified. Most importantly, the influence of NKAα1 on autophagy in nervous system, especially under ischemic conditions, has not been investigated.

To investigate the potential relationship between NKAα1 and autophagy in ischemia, a stable NKAα1 knockout (KO) N2a cell line was generated using Na^+^/K^+^ ATPase-α1 CRISPR/Cas9 KO plasmids (Fig. S1a). We examined the autophagy level in cells subjected to oxygen glucose deprivation/reperfusion (OGD/R) model, a widely used cell model that mimics cerebral ischemic insult. BafA1 (100 nM) was added to each group upon reperfusion to magnify and visualize autophagy flux by evaluating accumulated LC3II levels. We found that NKAα1 loss impaired autophagy in N2a cells under both normoxia and hypoxia, as shown by reduced LC3II levels in the presence of BafA1 (Figs. 1a and S1c). With quantitative polymerase chain reaction (qPCR), we measured the mRNA levels of autophagy-related proteins in both WT and NKAα1 KO cells. All the mRNAs tested were significantly reduced in NKAα1-deficient cells (Fig. 1b). Similar results were obtained in the brain cortex from NKAα1^+/+^ and NKAα1^+/−^ mice (Fig. S1d). These findings reveal the critical role of NKAα1 in autophagy regulation in brain physiology.

**Fig. 1:**
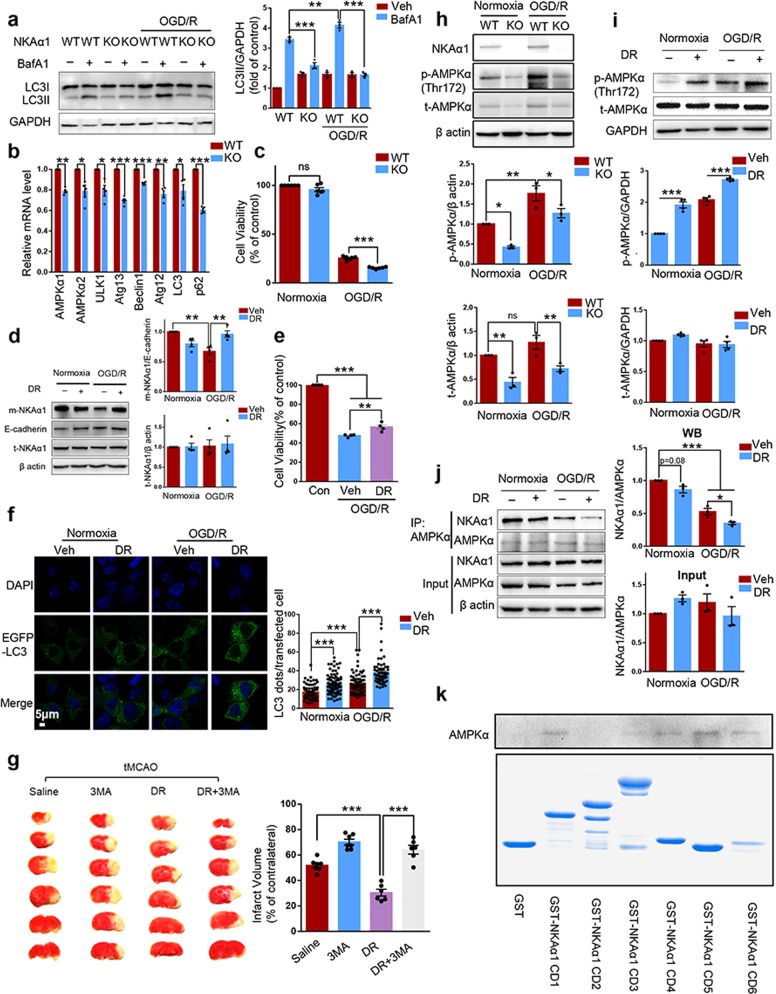
**a** Western blots showing that OGD/R-enhanced autophagy in WT cells was abolished in NKAα1 KO cells. Both WT and NKAα1 KO cells were subjected to OGD (3 h)/R (2 h) in the presence or absence of BafA1 (100 nM). *n* = 3. **b** qPCR analysis showing that the mRNA levels of autophagy-related proteins (AMPKα1, AMPKα2, ULK1, Atg13, Beclin1, Atg12, LC3, and p62) were significantly reduced in NKAα1 KO cells compared with WT N2a cells. *n* = 4. Atg13 Autophagy-related protein 13, Atg12 Autophagy-related protein 12. **c** Cell viability assay showing that NKAα1 loss exacerbated OGD/R-induced damage. *n* = 6. **d** Western blot analysis showing that DR-Ab treatment reversed the loss of membrane NKAα1 caused by OGD/R, while the total level of NKAα1 remained unaffected in response to OGD/R model. *n* = 4, DR DR-Ab, Veh vehicle, m-NKAα1 membrane NKAα1, t-NKAα1 total NKAα1. **e** Cell viability test showing the protective effect of DR-Ab. *n* = 4. Con control, Veh vehicle, DR DR-Ab. **f** Confocal microscopy images showing that DR-Ab significantly increased LC3 dots in N2a cells transfected with EGFP-LC3 plasmid under both normoxic and hypoxic conditions. BafA1 (100 nM) was added to each group to magnify and visualize autophagy flux. Magnification: 300×. LC3 dots were counted with ImageJ software. Data were from three independent experiments. A total of 55–110 cells were counted in each group. Scale bar: 5 μm. **g** Representative TTC-stained brain sections and quantitative data showing that the blockade of autophagy with 3MA abolished the protective effect of DR-Ab on the infarction volume caused by tMCAO. *n* = 6–7/group. 3MA 3-methyladenine, 100 nM/mouse, 2 h before ischemia, icv; DR: DR-Ab, 200 μg/mouse, 1 h before ischemia, iv; DR + 3MA: (3MA, 100 nM/mouse, 2 h before ischemia, icv) + (DR-Ab, 200 μg/mouse, 1 h before ischemia, iv). **h** Western blot analysis showing that NKAα1 KO reduced total and phosphorylated AMPKα levels under both normoxic and hypoxic conditions. *n* = 3. **i** Western blot analysis showing that DR-Ab significantly increased the levels of AMPKα phosphorylated at Thr172 under both normoxic and hypoxic conditions. *n* = 3–4. p-AMPKα phosphorylated AMPKα, t-AMPKα total AMPKα. **j** Representative western blots showing that OGD/R induced the dissociation of NKAα1 and AMPKα, which was further enhanced by DR-Ab treatment. Cells were immunoprecipitated with anti-AMPKα antibody, followed by NKAα1 antibody detection. *n* = 3. **k** In vitro GST pull down assay to define the specific intracellular domains of NKAα1 responsible for its direct interaction with AMPK. The purity and amount of purified GST-fused protein were shown in the lower panel. ns not significant, *p < 0.05, **p < 0.01, ***p < 0.001 as indicated. Bars represent the mean ± s.e.m. Unpaired two-tailed *t*-test (**b**) or one-way ANOVA with Bonferroni’s multiple comparison test (**a**, **c**, **d**, **e**, **f**, **g**, **h**, **i**, and **j**)

NKAα1 KO cells were more susceptible to OGD/R-induced cell injury, as reflected by cell viability assay (Fig. 1c). We further examined the effect of OGD/R on NKAα1 expression. No obvious change in total NKAα1 was detected while its membrane expression was largely reduced in response to OGD/R (Fig. 1d). We previously reported an antibody named DR-Ab targeting the DR region of NKAα1 in the 4th extracellular domain (897DVEDSYGQQWTYEQR911) protected cells against glutamate-induced excitotoxicity.^5^ In this study, our data indicate that purified DR-Ab treatment rescued the loss of membrane NKAα1 caused by hypoxia (Fig. 1d) and mitigated OGD/R-induced cell death (Figs. 1e and S2b). The contribution of autophagy to the cytoprotective effect of DR-Ab against hypoxia was determined thereafter. DR-Ab treatment enhanced LC3II expression and SQSTM1/p62 (a substrate of autophagy) degradation in response to hypoxia induction (Fig. S3a, b). Consistently, DR-Ab also enhanced LC3 dot accumulation in cells transfected with EGFP-LC3 plasmid (Fig. 1f). Moreover, cell viability assay showed that the protective effect of DR-Ab was dramatically attenuated upon autophagy inhibition by 3MA (3-methyladenine) treatment (Fig. S3c). Taken together, our data suggest that the protective effect of DR-Ab is, at least partially, mediated by the modulation of autophagy.

The therapeutic effect of DR-Ab in vivo was studied by its intravenous (iv) injection into mice subjected to transient middle cerebral artery occlusion (tMCAO) model. Immunofluorescent staining showed that DR-Ab was able to pass through the blood brain barrier after tMCAO surgery (Fig. S4a). 2,3,5-Triphenyltetrazolium chloride staining revealed that DR-Ab treatment both before and after tMCAO surgery successfully relieved brain infarction (Fig. S4b, d). In addition, neuronal loss in the penumbra area caused by tMCAO was also significantly ameliorated by DR-Ab (Fig. S4c). We also studied the involvement of autophagy in the protective effects of DR-Ab in vivo. Cortical tissues from the penumbra area in the brains of tMCAO mice and corresponding sham-operated mice were dissected for protein expression analysis. The phosphorylation of ULK1 and the protein expression of LC3II were both significantly increased in tMCAO mice compared with sham-operated mice, and these effects were further reinforced by DR-Ab (Fig. S5). Intracerebroventricular administration of 3MA abolished the protective effects of DR-Ab (Fig. 1g). In general, the above data indicate that autophagy contributes to the protective effect of DR-Ab against ischemic injury in vivo.

The levels of AMPKα, one of the most important positive regulators of autophagy, was lower in the brain cortex of NKAα1^+/−^ mice than in NKAα1^+/+^ mice (Fig. S6a), implying that normal operation of AMPK protein requires sufficient NKAα1 expression. This was further confirmed in NKAα1 KO cells, in which both total and phosphorylated AMPK levels were dramatically reduced (Fig. 1h). This explains the reduced autophagy flux in NKAα1 KO cells. AMPK pathway was activated in OGD/R and further enhanced by DR-Ab treatment (Figs. 1i and S6b, c). Blockade of AMPK with compound C reversed the protective effect of DR-Ab on OGD/R-induced cell death, as indicated by PI staining (Fig. S6d).

Cell lysate was collected to perform co-immunoprecipitation analysis. A direct interaction between AMPK and NKAα1 was found and this interaction was disrupted by hypoxia insult. This hypoxia-induced disruption was further enhanced by DR-Ab treatment (Fig. 1j). The above thrilling observation implies that the direct interaction between AMPK and NKAα1 under physiological conditions may impede the activation of AMPK and thus maintain relatively low autophagy levels. Disruption of certain binding during starvation seemed to account for the active phosphorylation of AMPKα at Thr172 residue and subsequent autophagy induction. A conformational change in NKAα1 caused by DR-Ab binding might turn this large transmembrane protein to adopt a new conformation unsuitable for the interaction with AMPK, which in turn activating abundant AMPK and causing a surge in autophagy flux. An in vitro glutathione S-transferase (GST) pulldown assay was then performed to examine the specific cytoplasmic domains of NKAα1 responsible for the interaction with AMPK. AMPKα was pulled down by all the GST-NKAα1 intracellular domain-fused proteins except the second one (Fig. 1k). We therefore concluded that multiple interaction sites between these two proteins exist. However, our work is limited to the lack of demonstration of the conformational change in NKAα1 upon DR-Ab treatment. More experiments are also warranted to clarify the machinery responsible for the dissociation of NKAα1 and AMPK in response to hypoxia induction.

In summary, our data demonstrated that NKAα1 and AMPK may serve as a switch accounting for the “on” and “off” states of autophagy. This study unveils a novel mechanism by which NKAα1 acts as a signal transducer and mediator of autophagy during ischemia-reperfusion and offers new insight into the interventions for ischemic stroke.

## Supplementary information

Supplementary Materials for Na+/K+-ATPase-dependent autophagy protects brain against ischemic injury
