# Supplementary material for: Na+/K+-ATPase-dependent autophagy protects brain against ischemic injury
Source: Signal Transduct Target Ther. 2020 May 20;5:55. doi: 10.1038/s41392-020-0153-7 (PMC7237650; doi:10.1038/s41392-020-0153-7)
Supplement: Supplementary file 1 — Supplementary Materials for Na+/K+-ATPase-dependent autophagy protects brain against ischemic injury [file 41392_2020_153_MOESM1_ESM.docx]

Supplementary Materials for

Na^+^/K^+^-ATPase-dependent autophagy protects brain against ischemic injury

Mengyuan Zhu^1^, Lei Cao^1^, Siping Xiong^1^, Haijian Sun^1^, Zhiyuan Wu^1^, Jin-Song Bian^1,2*^

*Correspondence to Dr. Jin-Song Bian: [phcbjs@nus.edu.sg](mailto:phcbjs@nus.edu.sg)

**This PDF file includes:**

Materials and Methods;

References for ‘Materials and Methods’;

Figure. s1-s8.

# Materials and methods

## Chemicals and reagents

LC3B antibody (ab48394), pospho-ULK1 (S556) antibody (ab203207), NeuN antibody (ab177487) were purchased from Abcam. Phospho-AMPKα (Thr172) antibody (#2531), AMPKα antibody (#2532), ULK1 antibody (#8054), cleaved caspase3 antibody (#9661) were purchased from Cell Signaling Technology. NKAα antibody (sc-48345), NKAα1 antibody (sc-21712), β actin (sc-81178), GAPDH (sc-32233), E-cadherin (sc-8426) were purchased from Santa Cruz. Bafilomycin A1 (SML1661), 2,3,5-Triphenyltetrazolium chloride (TTC) (T8877), Cell Counting Kit-8 (96992) were purchased from Sigma. EZ-Link^TM^  Sulfo-NHS-SS-Biotin (#21331), Pierce Streptavidin Ultra Link Resin (#53114), Glucose free DMEM (#11966025), Pierce^TM^ Glutathione Agarose (#16100), Pierce^TM^ GST Spin Purification Kit (#16107) were purchased from Thermo Fisher Scientific.

## NKAα1 KO stable Neuro-2a (N2a) cell line generation

NKAα1 CRISPR/Cas9 KO plasmid (sc-419236) was bought from Santa Cruz. NKAα1 KO stable N2a cell line was generated according to company’s instructions. Briefly, cells were transfected with NKAα1 CRISPR/Cas9 KO Plasmid containing three plasmids encoding CRISPR-associated protein (Cas9 nuclease) and three different 20nt gRNA (Sequence-1 (Sense): GTATGAGCCTGCCGCTGTAT; Sequence-2 (Sense): GTCACCATGCT CCGATAC AG; Sequence-3 (Sense): AATCTGTTCCGTATTTACGA) targeting NKAα1 gene for high KO efficiency. HDR plasmid was co-transfected in order to repair site specific double strand breaks (DSB) caused by KO plasmids. Cells went through DSB and subsequent repairment incorporates puromycin resistance and RFP fluorescence which can be used to confirm successful transfection. Positive clones were picked and the efficiency of NKAα1 KO was determined by Western blotting.

## Quantitative polymerase chain reaction (qPCR)

Total cellular RNA from N2a cells were isolated and reverse transcription kit was applied afterwards to generate cDNAs (Promega, A5001). 1μg cDNA was used in 10 μl SYBR Green-based real-time PCR for quantification of mRNA level (Promega, A6001). Specific primers in this experiment were listed as below. AMPKα1, F: 5’-CTCAGTTCCTGGAGAA AGATGG-3’, R: 5’-CTGCCGGTTGAGTATCTTCAC-3’; AMPKα2, F: 5’- CATGGCTGAG AAGCAGAAGCAC-3’, R: 5’- CTTAACTGCCACTTTATGGCCTG-3’; ULK1, F: 5’- CACT GCGTGGCTCACCTAAG-3’, R: 5’- AGCCAACAGGGTCAGCAAAT-3’; Atg13, F: 5’- GTGGGCACCCTCACTCTTTC-3’, R: 5’- GGGATAGGGACGGTCAACAA-3’; Beclin1, F:5’- CTTGGAGGAGGAGAGGCTGA-3’, R: 5’- TGTGGAAGGTGGCATTGAAG-3’; Atg12, F: 5’- GGCCTCGGAACAGTTGTTTA-3’, R: 5’- CAGCACCGAAATGTCTCTGA-3’; MAP1LC3B/LC3, F: 5’- TTATAGAGCGATACAAGGGGGAG-3’, R: 5’- CGCCGTCTGAT TATC TTGATGAG-3’; SQSTM1/p62, F: 5’- AGGCGCACTACCGCGAT-3’, R: 5’- CGTCACTGGA AAAGGCAACC-3’;β-actin, F: 5’- CCGTGAAAAGATGACCCAGA-3’, R: 5’- TACGACCAGAGGCATACAG-3’.

## Oxygen Glucose Deprivation/ Reperfusion model (OGD/R)

The method of OGD/R was described before [^1^](#_ENREF_1). In brief, cultures were rinsed twice with warm PBS, and then refreshed with glucose-free DMEM (Thermo Fisher Scientific, 11966025). Cells were then immediately placed in a sealed chamber (Billups Rothenburg, 5352414) loaded with mixed gas containing 5% CO2 and 95% N2 for 5min at 10 L/min. Cells were incubated at 37°C for 3h before reperfusion. For reperfusion, cells were refreshed with normal culture medium and incubated for another 2h in 37°C incubator.

## Cell viability assay

Cell viability was examined by cell count kit (Sigma, 96992) according to manufacturer’s instructions. Briefly, cells were pre-treated with 10μg/ml purified DR-Ab for 2h. After that, cells were subjected to OGD(3h) /R(2h). Upon completion of OGD/R, cell medium was refreshed with 100μl fresh DMEM and incubate for another 3h at 37°C. 10ul CCK8 detection solution was added into each well at the completion of reperfusion. The absorbance at 450nm was measured using a microplate reader. Results were expressed as relative cell viability (% of control).

## Transient Middle Cerebral Artery Occlusion (tMCAO)

C57BL6 mice (male, 8 weeks, n≥12 per group) were subjected to tMCAO surgery as described [^2^](#_ENREF_2). Briefly, mice were anesthetized with 5% isoflurane. Focal cerebral ischemia was induced by occlusion of the middle cerebral artery through insertion of MCAO monofilament (Doccol, 602256PK10). Reperfusion was allowed 1h after occlusion through removal of the monofilament and animals were sacrificed 24h after reperfusion. Purified DR-Ab (200μg/mouse) or normal IgG purified from normal serum were administrated by *iv* injection 1h before or after surgery and 3MA (100nM/mice) was administrated by stereotaxic injection into right lateral ventricle (1 mm ML, -0.22 mm AP from bregma, 2.3 mm below the dura) 2h before occlusion. Brain was harvested 24h after reperfusion and stained using TTC (SIGMA, T8877) to evaluate the infarction volume. The equation for edema corrected infarction ratio was indicated as below: (volume of contralateral hemisphere − volume of non-lesioned area in ipsilateral hemisphere)/volume of contralateral hemisphere × 100%. All the procedures involving animal work were proved by Institutional Animal Care and Use Committee (IACUC), National University of Singapore.

## Biotinylation of cell surface proteins

Cells cultured in petri dishes were placed on ice and rinsed with pre-cold PBS once. Membrane proteins were labelled with 1 mg/ml EZ-link NHS-SS-biotin (Thermo Fisher Scientific, 21331) in PBS as described before [^3^](#_ENREF_3). After 1h incubation, cells were washed by PBS containing 100 mM glycine for three times and subsequently lysed in modified RIPA buffer (50mM Tris- HCl, pH 8; 150mM NaCl; 1% Triton X-100 and 1% sodium deoxycholate; 10 μg/ml leupeptin; 100 μg/ml TPCK; 1mM PMSF). Equal proteins (300 μg) for each group were incubated together with equivalent Streptavidin beads (Thermo Fisher Scientific, 53114) overnight at 4 °C. Beads were washed several times to remove unspecific binding, resuspended in 40 μl 2X loading buffer and kept for Western blotting analysis.

## GST pull-down assay

Six pGEX-6p-1 plasmids containing sequences for cytoplasmic domains of NKAα1 were kindly provided by Dr. Emilia Lecuona in Northwestern University, USA, as a gift [^4^](#_ENREF_4). These six GST fusion proteins were expressed in BL21 bacterial cells and purified by Pierce^TM^ GST Spin Purification Kit (Thermo Fisher Scientific, 16107) according to its protocol. Size and purity of these proteins were determined by Coomassie blue staining. Equal cell lysates were incubated with proteins bound to glutathione beads overnight at 4°C with end-over-end shaking. Beads were rinsed with PBS several times, resuspended in loading buffer and analysed by western blotting.

## Co-immunoprecipitation assay

Cells were lysed with lysis buffer on ice completely. 500μg protein from each group were then incubated with anti-AMPKα antibody at 4°C for 1h. 20μl Protein G Agarose were then added and incubate overnight at 4°C. Beads were rinsed several times with PBS and resuspended with 2X loading buffer, separated and analysed by 12% SDSPAGE.

## Immunohistochemistry (IHC)

The protocol for IHC has been clarified before [^5^](#_ENREF_5). Mice(n≥3) were anesthetized and perfused with PBS followed by 4% paraformaldehyde. Brains were then incubated in 4% paraformaldehyde overnight at 4 °C followed by dehydration with increasing gradient concentration of sucrose solution, mounted in OCT embedding compound and freeze at -20 °C. Coronal sections from frontal to occipital direction were sliced using a cryostat at a thickness of 30μm. 1% H_2_O_2_ was applied to block endogenous peroxidase activity for 30min at room temperature. Brain slices were then rinsed with PBS and blocked with 5% BSA in PBS (containing 0.3%Triton X-100 for permeabilization) for another 1h. Primary antibody rabbit monoclonal anti-NeuN (Abcam, 1:500) was added and mildly shaking overnight at 4 °C. After several wash in PBS, sections were incubated with horseradish peroxidase (HRP) goat anti-rabbit secondary antibody for 1h. These slices were then visualized with 3,3'-diaminobenzidine (DAB) (BOSTER, AR1022). Images were captured with TissueFAXS Slide Scanner and number of NeuN-positive cells in penumbra were blindly counted using Image-Pro Plus 6.0 software.

For immunofluorescence staining of rat DR-Ab across blood brain barrier, Alexa Fluor 488 goat anti-rat secondary antibody was applied on brain slice to visualize the permeability of DR-Ab under fluorescence microscopy.

## GFP-LC3 dot assay

N2a cells were transiently transfected with EGFP-LC3 plasmid using Lipofectamine 3000 transfection kit (Invitrogen, L3000015) according to manufacturer’s instructions. After at least 24h, cells were subjected to OGD/R model following purified DR-Ab and BafA1 treatment as indicated before and observed under confocal microscope. To quantify accumulated LC3 dots following OGD/R, at least six fields in each group were captured in a single experiment and three independent experiments were performed. More than 55 cells in each group were used for final quantification.

## Antibody generation and purification

Detailed procedure for DR-Ab generation and purification was illustrated in our previous publication [^6^](#_ENREF_6). In general, rats were treated with keyhole limpet hemocyanin (KLH) conjugated DR peptide (897DVEDSYGQQWTYEQR911) subcutaneously every two weeks with an initial dose of 200μg protein emulsified with complete Freund’s adjuvant (CFA) followed by 100μg protein emulsified with incomplete Freund’s adjuvant (IFA) for three times. Serum of immunized rats were collected and was purified with protein A/G spin column (Thermo Fisher Scientific, 89962).

## Western blotting

Equal amount of protein samples was separated by 8%-15% SDSPAGE and transferred to PVDF membrane. Membrane was blocked in TBST containing 5% milk for 1h and then probed with specific primary antibody at 4°C overnight. After washed three times, secondary antibody was added and incubate for 1-2h in room temperature. Chemiluminescence detection kit (Millipore, WBKLS0500) was applied for final visualization. For quantification of protein expression, densitometric analysis was performed using Image-Pro Plus software.

## Statistics

Data were presented as mean ± SEM. One-way ANOVA were applied for comparisons among groups where appropriate. Post-hoc comparisons were achieved by Bonferroni’s multiple comparisons test. For comparisons between two groups, Student’s unpaired two tailed t test were employed. p < 0.05 was regarded statistically significant. Bars represent mean ± s.e.m.

## Data Availability

The derived data used to support the findings of this study are included within the article and are available from the corresponding author upon request.

**References**

1 Shi, Z. *et al.* Protection against Oxygen-Glucose Deprivation/Reperfusion Injury in Cortical Neurons by Combining Omega-3 Polyunsaturated Acid with Lyciumbarbarum Polysaccharide. *Nutrients*. **8**, (2016).

2 Longa, E. Z., Weinstein, P. R., Carlson, S. & Cummins, R. Reversible middle cerebral artery occlusion without craniectomy in rats. *Stroke*. **20**, 84-91, (1989).

3 Lecuona, E. *et al.* The GTP-binding protein RhoA mediates Na,K-ATPase exocytosis in alveolar epithelial cells. *Mol Biol Cell*. **14**, 3888-3897, (2003).

4 Lecuona, E. *et al.* Na,K-ATPase alpha1-subunit dephosphorylation by protein phosphatase 2A is necessary for its recruitment to the plasma membrane. *FASEB J*. **20**, 2618-2620, (2006).

5 Wang, M. *et al.* Compound porcine cerebroside and ganglioside injection attenuates cerebral ischemia-reperfusion injury in rats by targeting multiple cellular processes. *Neuropsychiatr Dis Treat*. **13**, 927-935, (2017).

6 Shi, M. *et al.* DR-region of Na(+)/K(+) ATPase is a target to treat excitotoxicity and stroke. *Cell Death Dis*. **10**, 6, (2018).

**
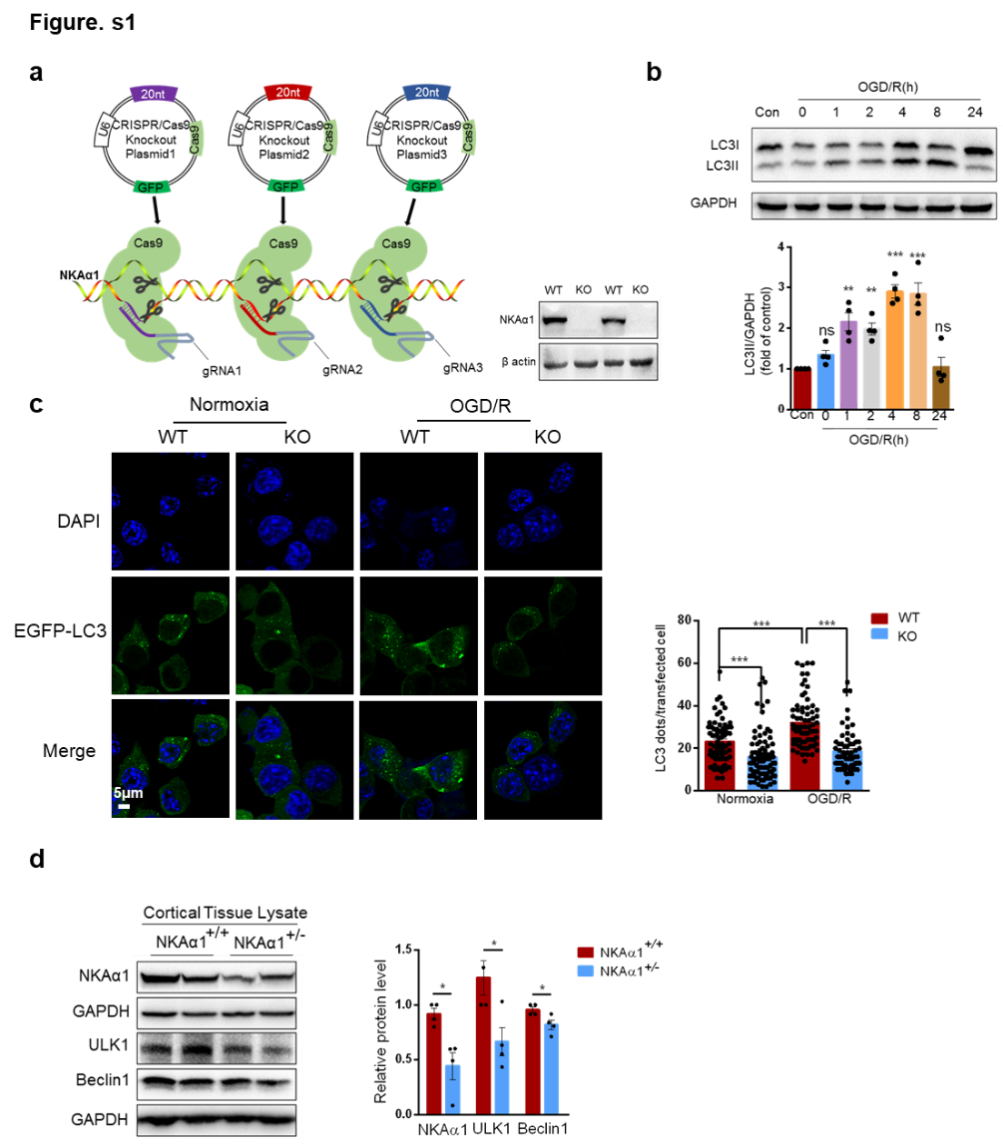
**

Figure. s1 (**a**) Schematic illustration showing the generation of NKAα1 KO stable cell line. The efficiency of NKAα1 KO was determined by Western blotting. (**b**) Western blots showing the autophagy induction upon OGD for 3h and reperfusion for different time point. n=4. (**c**) Autophagosome counting under confocal microscopy after cells transfected with EGFP-LC3 plasmid suggests that autophagy level was significantly decreased in NKAα1 deficiency cells in hypoxia. BafA1(100 nM) was added in each group to magnify and visualize autophagy flux. Magnification: 300x. LC3 dots were counted with ImageJ software. More than 60 cells were counted in each group. Scale bar: 5 μm. (**d**) Western blotting analysis showing that the expression of ULK1, Beclin1 were decreased in NKAα1^+/-^ mice brain cortex compared to that in WT mice. n=4. ns, not significant, *p<0.05, **p<0.01, ***p<0.001 as indicated. Bars represent mean ± s.e.m. Unpaired two-tailed t-test (d) or one-way ANOVA with Bonferroni's multiple comparisons test (b, c).

**
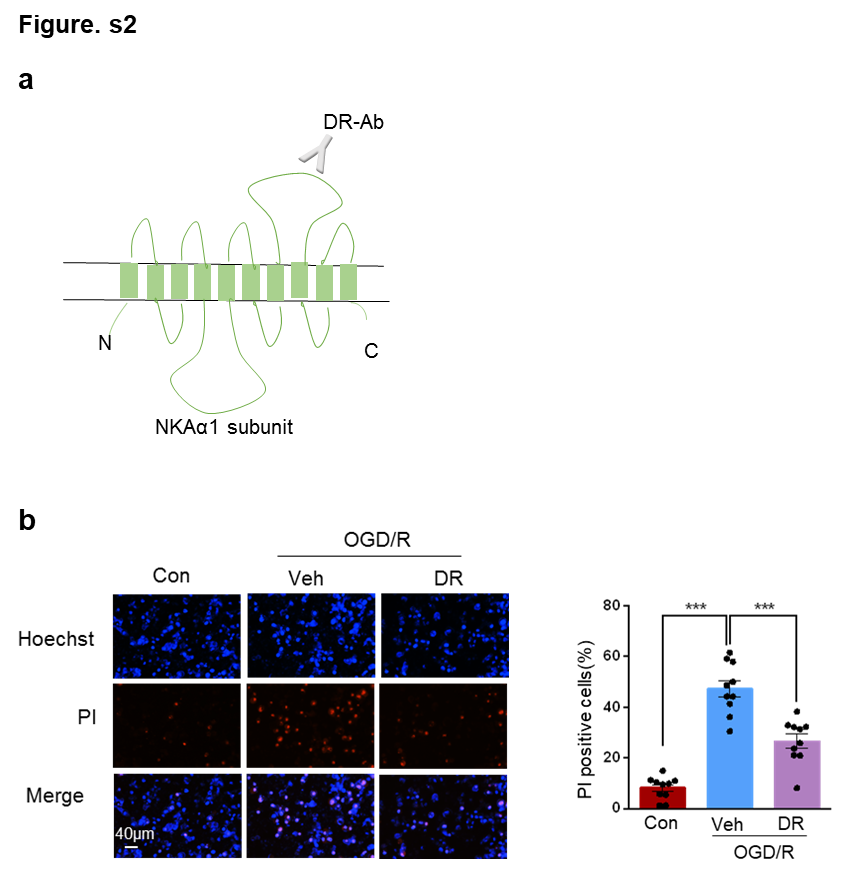
**

Figure. s2 (**a**) Schematic illustration describing DR-Ab binding site at the fourth extracellular domain of NKAα1. (**b**) Propidium Iodide (PI) staining showing that DR-Ab treatment reversed cell apoptosis caused by OGD/R. scale bar: 40 μm. Data was presented as PI/Hoechst stained cell (%). n=3, at least 9 fields observed for each group in total. Con: control; Veh: Vehicle; DR: DR-Ab. ***p<0.001 as indicated. Bars represent mean ± s.e.m. One-way ANOVA with Bonferroni's multiple comparisons test (b).

**
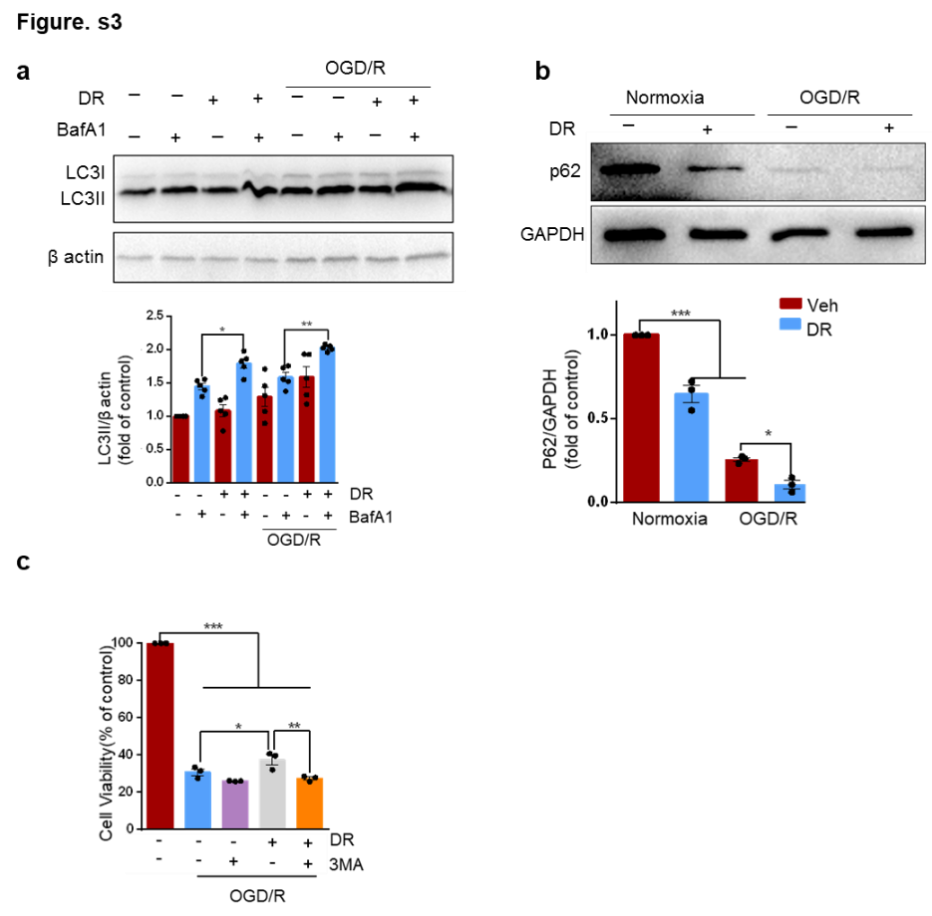
**

Figure. s3 (**a-b**) Western blotting analysis showing that DR-Ab treatment enhanced LC3II expression (**a**, n=5) and accelerated the degradation of substrate SQSTM1/p62 (**b**, n=3). (**c**) Cell viability assay showing that blockade of autophagy abolished the protective effect of DR-Ab(10μg/ml) against OGD/R-induced cell damage. n=3. 3MA: 3-methyladenine, 5mM, added 3h before OGD. *p<0.05, **p<0.01, ***p<0.001 as indicated. Bars represent mean ± s.e.m.One-way ANOVA with Bonferroni's multiple comparisons test (a, b, c).

**
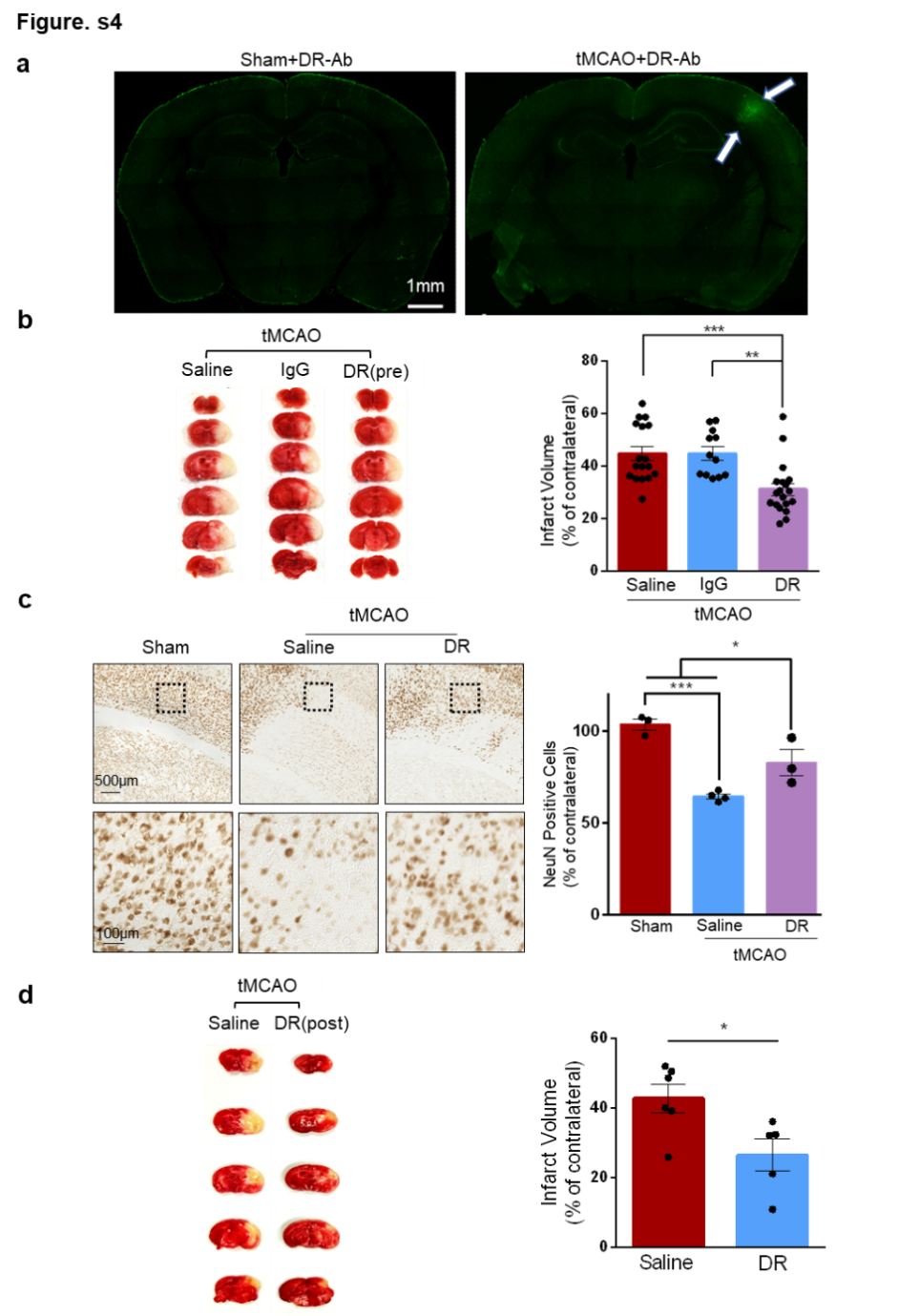
**

Figure. s4 (**a**) Representative immunofluorescence staining showing the permeability of DR-Ab across BBB in tMCAO mice into ipsilateral cortex but not in sham-operated mice. DR-Ab (200μg/mouse) purified from immunized rat serum was administrated by *iv* injection 1h before surgery. Coronal brain sections were sliced with cryostat. Alexa Fluor 488 goat anti-rat secondary antibody was applied to detect DR-Ab in brain slices. Scale bar: 1mm. (**b**) Representative TTC staining of brain slices and quantitative data showing that DR-Ab reduced tMCAO-induced infarction volume in the brain. DR-Ab was injected by tail vein 1h prior to occlusion. n=12-19. (**c**) Representative image and group data showing that DR-Ab attenuated the neuron loss in the penumbra area in the brain slice of tMCAO mice. Neurons were detected with NeuN antibody (visualized by DAB). n=3-4/group. (**d**) Representative TTC staining of brain slices and quantitative data showing that DR-Ab reduced tMCAO-induced infarction volume in the brain. DR-Ab was injected by tail vein 30min after reperfusion. n=5-6. *p<0.05, **p<0.01, ***p<0.001 as indicated. Bars represent mean ± s.e.m. Unpaired two-tailed t-test (d) or one-way ANOVA with Bonferroni's multiple comparisons test (b, c).

**
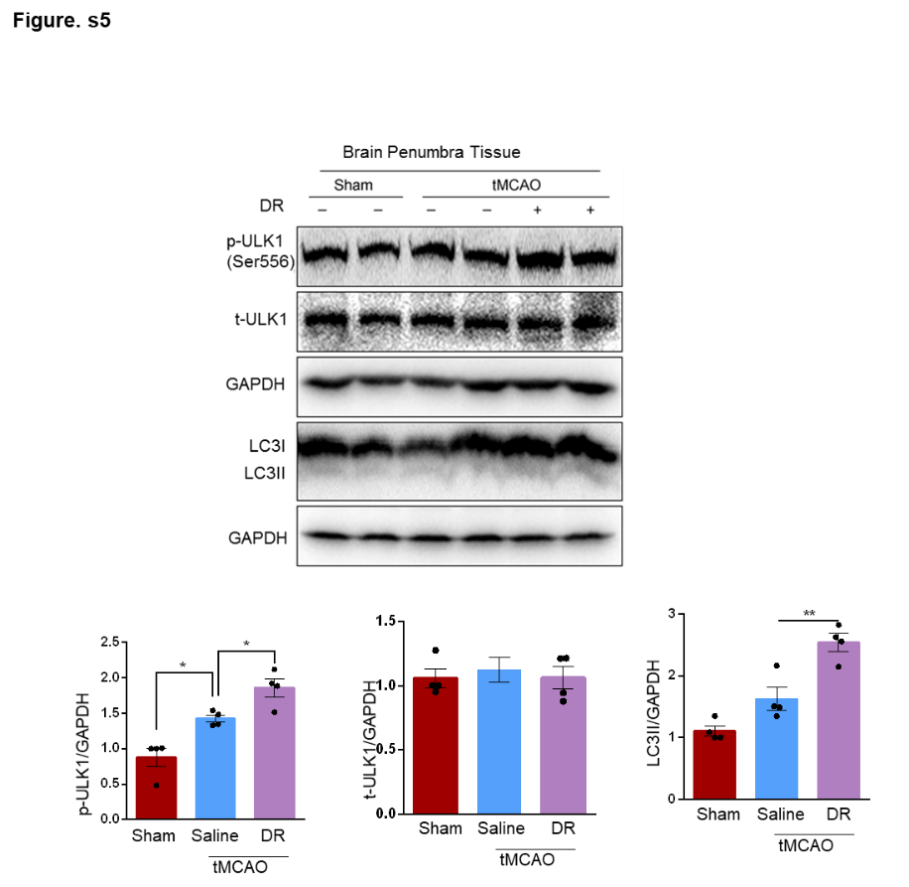
**

Figure. s5 Western blotting analysis showing that DR-Ab further enhanced tMCAO-induced p-ULK1 and LC3II expression. Brain penumbra fractions were dissected from mice subjected to tMCAO surgery. Corresponding cortex area from sham-operated mice treated with normal saline was regarded as control. n=4. *p<0.05, **p<0.01, as indicated. Bars represent mean ± s.e.m. One-way ANOVA with Bonferroni's multiple comparisons test.

**
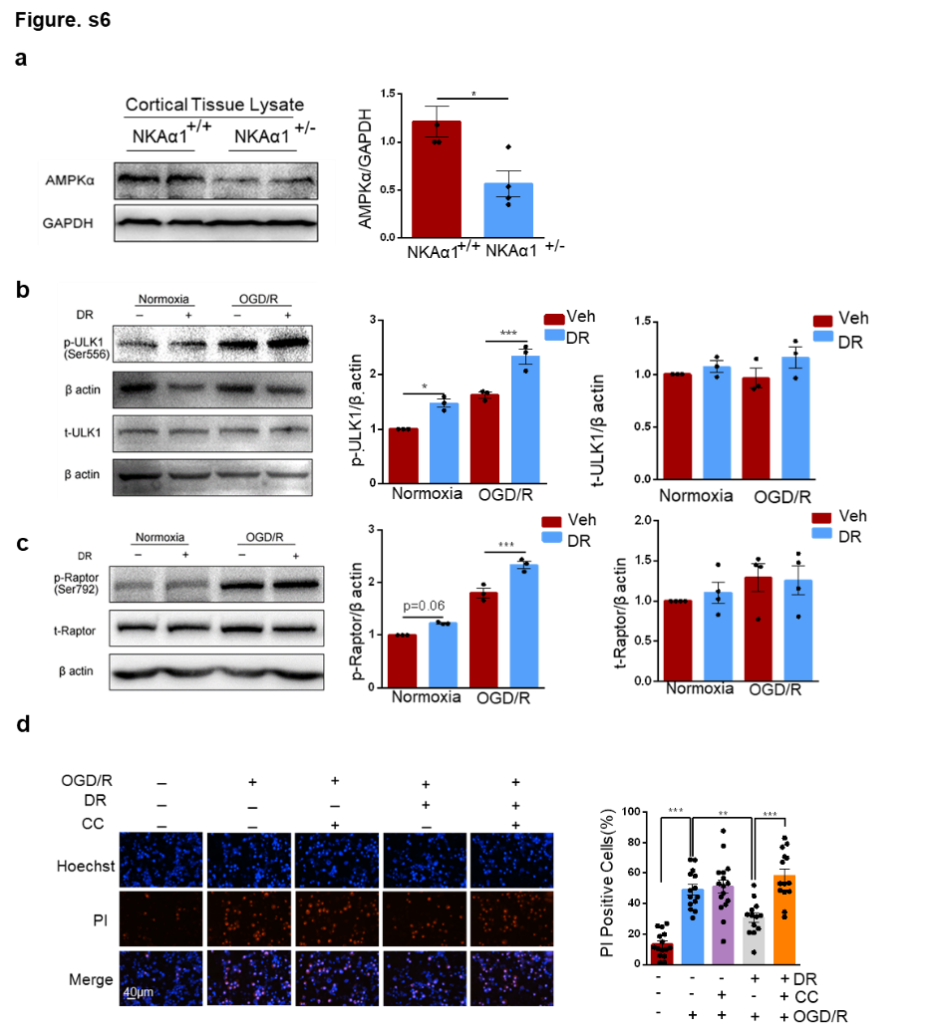
**

Figure. s6 (**a**) Western blotting analysis showing that AMPKα expression was largely reduced in the brain cortex of NKAα1^+/-^ mice when compared with those in NKAα1^+/+^ mice. n=4. (**b-c**) Western blotting analysis showing that DR-Ab significantly increased the phosphorylation levels of ULK1 at Ser555/556 residue (**b**) and Raptor at Ser792 residue (**c**) in both normoxic and hypoxic conditions. n=3-4. p-ULK1: phosphorylated-ULK1, t-ULK1: total-ULK1; p-Raptor: phosphorylated-Raptor, t-Raptor: total-Raptor. (**d**) Representative Hoechst 33342 and PI staining images and group data showing that blockade of AMPK with CC abolished the protective effects of DR-Ab on the apoptotic N2a cells. Scale bar: 40 μm. Data was presented as PI/Hoechst stained cells (%). n=3. At least 13 fields were observed in each group. DR: DR-Ab, CC: Compound C (AMPK inhibitor), 20μM, 3h before OGD/R model. *p<0.05, **p<0.01, ***p<0.001 as indicated. Bars represent mean ± s.e.m. Unpaired two-tailed t-test (a) or one-way ANOVA with Bonferroni's multiple comparisons test (b, c, d).

**
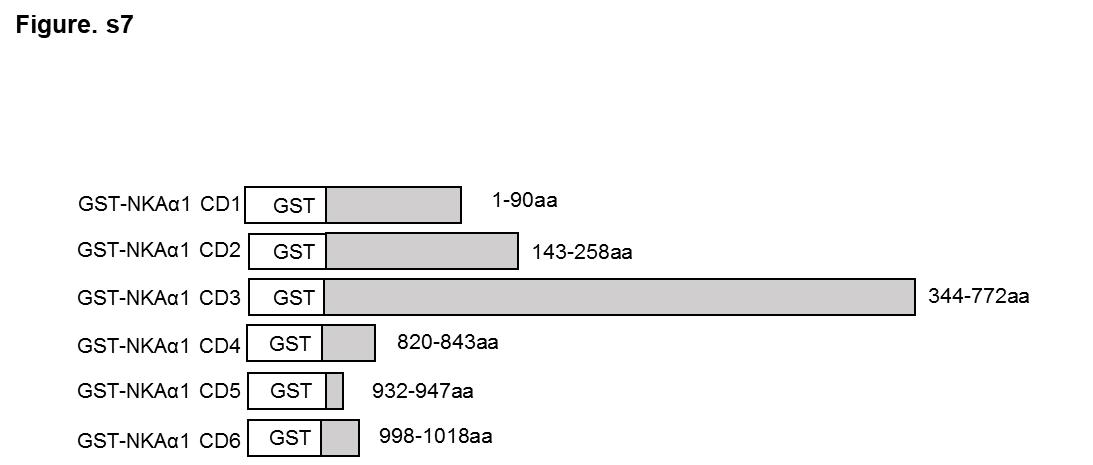
**

Figure. s7 Schematic diagram illustrating six pGEX-6p-1 plasmids containing sequences for cytoplasmic domains of NKAα1.

**
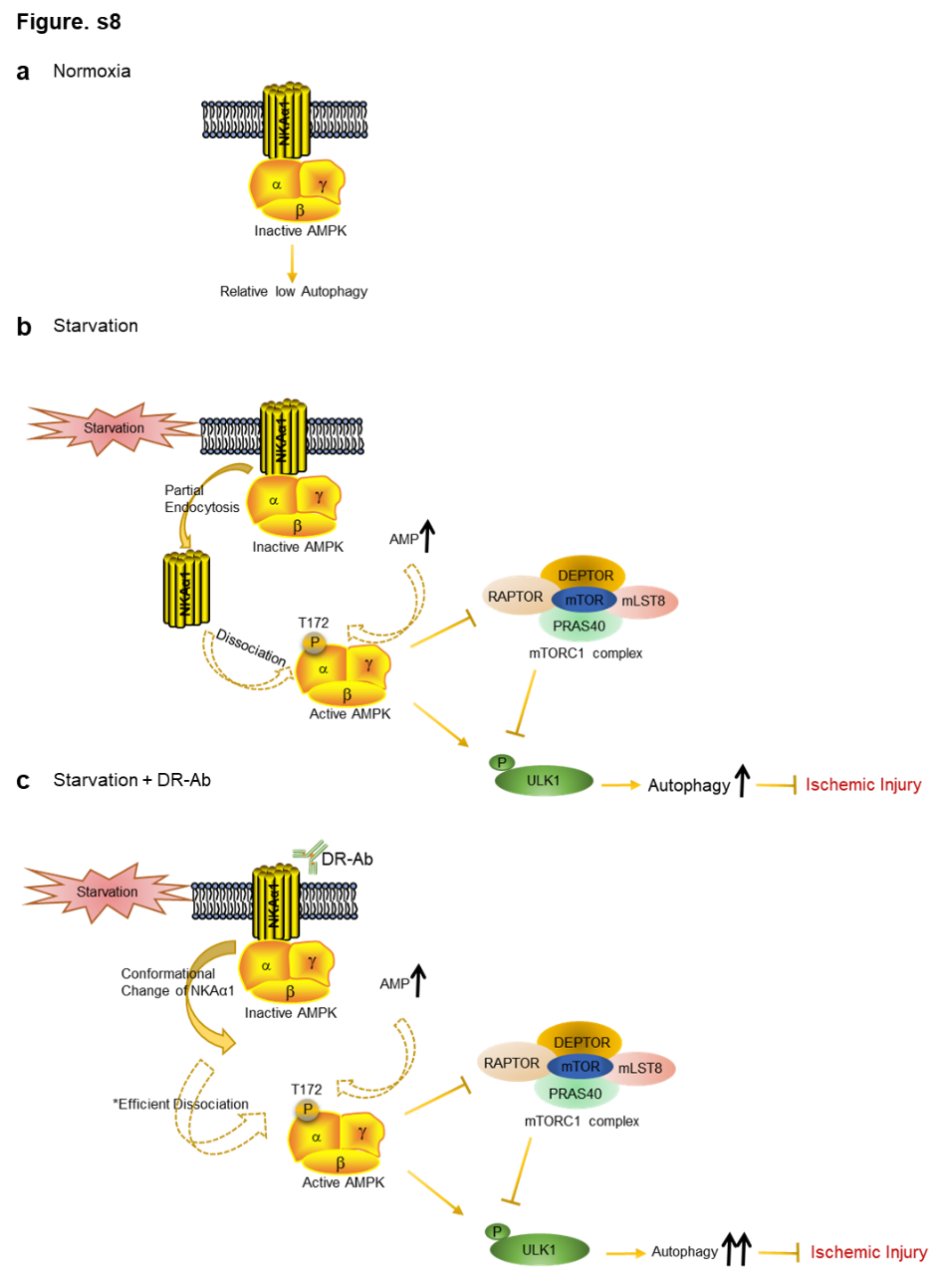
**

Figure.s8 Proposed mechanisms for NKAα1-dependent autophagy. (**a**) During quiescent condition, AMPK complex binds to membrane NKAα1 to promise relative low autophagy level for normal biological function. (**b**) When nutrient supply into brain is interrupted during ischemic stroke, endocytosis of NKAα1 leads to AMPK dissociation while total NKAα1 and AMPK level remains unchanged in short period. Elevated AMP thus rapidly activates functional AMPK, boosting active phosphorylation of ULK1 and inhibitory phosphorylation of mTORC1 complex which, in turn, initiates autophagy and eases ischemic injury. (**c**) Stabilization of NKAα1 upon DR-Ab binding during starvation ensures its normal pumping function. At the meantime, conformational change of membrane NKAα1 promotes the dissociation of AMPK and the subsequent departure from membrane and thus further activates AMPKα at Thr172 residue. The enhanced autophagy in this context protects neuronal cells against ischemic injury.
